# Children Show Highest Levels of Polybrominated Diphenyl Ethers in a California Family of Four: A Case Study

**DOI:** 10.1289/ehp.8554

**Published:** 2006-05-25

**Authors:** Douglas Fischer, Kim Hooper, Maria Athanasiadou, Ioannis Athanassiadis, Åke Bergman

**Affiliations:** 1 *Oakland Tribune*, Oakland, California, USA; 2 Environmental Chemistry Laboratory, California Department of Toxic Substances Control, Berkeley, California, USA; 3 Department of Environmental Chemistry, Stockholm University, Stockholm, Sweden

**Keywords:** BDE-209, brominated flame retardants, children, decaBDE, house dust, human exposure, PBDE, polybrominated diphenyl ethers

## Abstract

Polybrominated diphenyl ethers (PBDEs), a major class of flame retardants, are ubiquitous environmental contaminants with particularly high concentrations in humans from the United States. This study is a first attempt to report and compare PBDE concentrations in blood drawn from a family. Serum samples from family members collected at two sampling occasions 90 days apart were analyzed for PBDE congeners. Concentrations of the lower-brominated PBDEs were similar at the two sampling times for each family member, with children’s levels 2- to 5-fold higher than those of their parents. Concentrations of, for example, 2,2′,4,4′-tetrabromodiphenyl ether (BDE-47) varied from 32 ng/g lipid weight (lw) in the father to 60, 137, and 245 ng/g lw in the mother, child, and toddler, respectively. Decabromodiphenyl ether (BDE-209) concentrations differed significantly between the two samplings. September concentrations in the father, mother, child, and toddler were 23, 14, 143, and 233 ng/g lw, respectively. December concentrations (duplicate results from the laboratory) were 2 and 3, 4 and 4, 9 and 12, and 19 and 26 ng/g lw, respectively. Parents’ ∑PBDE concentrations approached U.S. median concentrations, with children’s concentrations near the maximum (top 5%) found in U.S. adults. The youngest child had the highest concentrations of all PBDE congeners, suggesting that younger children are more exposed to PBDEs than are adults. Our estimates indicate that house dust contributes to children’s higher PBDE levels. BDE-209 levels for all family members were 10-fold lower at the second sampling. The short half-life of BDE-209 (15 days) indicates that BDE-209 levels can decrease rapidly in response to decreased exposures. This case study suggests that children are at higher risk for PBDE exposures and, accordingly, face higher risks of PBDE-related health effects than adults.

The polybrominated diphenyl ethers (PBDEs) are brominated flame retardants sold as pentaBDE, octaBDE and decaBDE, containing mainly 4–5, 7–8, and 10 bromines, respectively, attached to the diphenyl ether moiety (Alaee et al. 2003). The PBDEs are added reportedly at 5–30% by weight to synthetic materials (e.g., polyurethane foams, synthetic fabrics, and thermoplastics) to retard ignition. PBDEs are non-covalently bound additives in a variety of consumer products, for example, TVs, computers, and fabrics, foams, and textiles used in homes, offices, and cars (Alaee et al. 2003; World Health Organization 1994, 1997). PBDEs are slowly released from these products during their life cycles (Alcock et al. 2003).

PBDE body burdens in the United States are relatively high: adult levels are 10–100 times higher than the 1–3 ppb body burdens found in Europe or Japan (Choi et al. 2003; Covaci et al. 2002; Mazdai et al. 2003; McDonald 2005; Petreas et al. 2003; Schecter et al. 2003, 2005b; She et al. 2002; Sjödin et al. 2001, 2003), and parts per million concentrations of PBDEs have been reported in human adipose tissue in New York City (Johnson-Restrepo et al. 2005). Levels of decaBDE (BDE-209) in humans are less widely measured but are found in several studies at 1–3 ng/g lipid weight (lw) (Hooper et al. 2004; Schecter et al. 2005b; She J, unpublished data; Sjödin et al. 2001; Thuresson et al. 2006). Several PBDE congeners have been shown experimentally to exert developmental neurotoxicity *in vivo* (Eriksson et al. 2002a, 2002b; Viberg et al. 2003, 2004). Some PBDEs also cause reproductive damage in laboratory animals at body burdens below 302 ng/g lw, a level reached by 5% of U.S. women (McDonald 2005).

To our knowledge, only one study has measured PBDE body burdens in the very young (Thomsen et al. 2002). The authors found PBDE levels in infants and children to be 2- to 3-fold higher than in adults. No study has compared PBDE levels among members of a family, despite evidence that PBDE exposures can differ significantly between age groups in a single household. For children, household dust is a major exposure pathway for many chemicals. ∑PBDE concentrations in household dust are 2–4 ppm (Rudel et al. 2003; Schecter et al. 2005a; Stapleton et al. 2005; Wilford et al. 2005), and toddlers may get 100-fold higher exposures to PBDEs from household dust than do adults (Jones-Otazo et al. 2005).

In addition, body burdens of PBDEs may change rapidly. For example, BDE-209 is a major (~ 50%) PBDE congener in household dust (Stapleton et al. 2005; Wilford et al. 2005) and has a half-life of 15 days in humans (Thuresson et al. 2006). Thus, BDE-209 levels have the potential to decrease rapidly after cessation of exposure, in contrast to the lower-brominated PBDEs, which have longer half-lives in years (Geyer et al. 2004; Sjödin et al. 2003). Our case report compares body burdens of PBDEs in children and adults and examines whether these concentrations change significantly over time.

## Materials and Methods

### Study design

We measured concentrations of PBDEs in two sets of serum samples collected 3 months apart from a family of four (35-year-old father, 36-year-old mother, 5-year-old daughter, and 18-month-old son) residing in Berkeley, California, USA. Samples were collected from each family member in mid-September 2004 and 90 days later in mid-December 2004 by a licensed phlebotomist at the same commercial clinical laboratory.

The family did not use common household cleaners and pesticides, had no wall-to-wall carpeting, and owned no large new appliances (Fischer 2005). The mother was a fourth-generation Californian who worked as a university researcher. The father was from the East Coast and taught high school. The daughter attended kindergarten, while the toddler son spent time at child care and at home. The toddler was exclusively breast-fed for 6 months and was breast-feeding during the study period. Family members gave informed consent prior to the study, and the study received institutional review board approval (Independent Review Consulting, Inc., Corte Madera, CA, USA).

### Preparation of serum samples

Blood samples were collected in red-top Vacutainers (Becton-Dickinson, Franklin Lakes, NJ, USA). Serum samples were prepared in the clinic by allowing blood samples to coagulate for 1 hr in a darkened room, centrifuging for 15 min at 1,000 × *g*, and decanting the supernatant into amber glass vials. Frozen serum samples were shipped to the laboratories and stored at −20°C until analysis. The September 2004 samples were shipped to AXYS Analytical Services Ltd, Sidney, British Columbia (Laboratory 1). The December 2004 samples were sent to the Department of Environmental Chemistry, Stockholm University, Stockholm, Sweden (Laboratory 2). All procedures in both laboratories were conducted under low light levels, using either amber glass vessels or aluminum foil to shield samples from ultraviolet and fluorescent light.

### Analysis

Laboratory 1 analyzed the September 2004 serum samples for 39 PBDE congeners using U.S. Environmental Protection Agency (EPA) Method 1614 (U.S. EPA 2003) and high-resolution gas chromatography/high-resolution mass spectrometry (HRGC/HRMS) with isotope dilution. Prior to extraction, each sample was spiked with a standard solution containing a suite of ^13^C-labeled PBDEs (BDE-28, −47, −99, −100, −153, −154, −183, −209). One serum sample from each family member was analyzed, accompanied by two corn oil method blanks. Only the background concentrations for BDE-209 were considerable and of importance for the quantification, but corrections for background levels were made for all the analytes. Internal standard recoveries ranged from 84 to 100% for all congeners.

Laboratory 2 analyzed the December 2004 samples for six congeners (BDE-47, −99, −100, −153, −154, −209), applying the cleanup method described by Hovander et al. (2000) using low-resolution GC/MS applying selected-ion monitoring of the bromide ions *m/z* 79 and 81. Cleanup was performed in the laboratory clean room. The methodological details are given elsewhere, as are the instrument parameters (Hovander et al. 2000; Sjödin et al. 1999, 2001; Thuresson et al. 2005). Background concentrations were below the limit of quantification (LOQ) except for BDE-47 as expressed below. Mean recovery of the internal standard was 96% (range, 84–107%).

## Results

**Figure 1:**
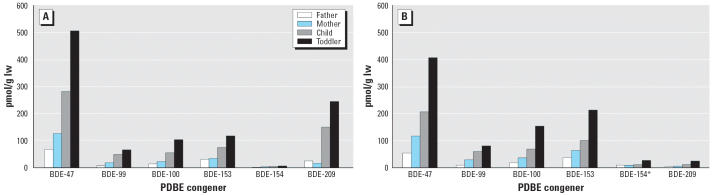
(*A*) PBDE concentrations (pmol/g lw) in September serum samples. (*B*) PBDE concentrations (pmol/g lw) in December serum samples. *May contain polybrominated biphenyl PBB-153.

Analytical results for the six major PBDEs are presented on a lipid weight basis in Table 1 (September samples by Laboratory 1, and December samples in duplicate by Laboratory 2) and on a molar basis in Figures 1A and 1B. The sums [∑(5)PBDE] of the five commonly reported PBDE congeners (BDE-47, −99, −100, −153, and −154) are also presented in Table 1. Recoveries of the analytes were between 84 and 100% for Laboratory 1 and 84 and 107% for Laboratory 2.

Laboratory 1 blank concentrations for the PBDE congeners 47, 99, 100, and 153 (4.7, 3.8, 0.8, and 0.7 pg/g corn oil, respectively) were small relative to measured levels. The mean sample blank concentrations were < 3% for the four PBDE congeners, except for BDE-99 in the father (6%). Laboratory 1 blanks for BDE-209 were higher (mean, 103 pg/g corn oil), with the highest blank (26 pg/g corn oil) relative to measured value [78 pg/g wet weight (ww)] occurring with the mother. Laboratory 2 blank concentrations for all PBDE congeners were under the LOQ < 0.1 ng/g lipids. The background level for BDE-47 was determined to be 0.1 ng/g lipids and neglected. Blank concentrations were only subtracted from Laboratory 1, and the corrected values are presented in Table 1.

## Discussion

Data from this case study of one family indicate that serum levels of PBDEs in this family differed significantly among different ages and between sampling times, and that PBDE levels are much higher in the infant and young child than in their parents. This contrasts with what would be expected with the classical persistent pollutants such as polychlorinated biphenyls and dichlorodiphenyldichloroethylene (DDE), which would show higher concentrations with age (Päpke 1998; Rylander et al. 1997). These results suggest that young children are at high risk of PBDE exposures.

Data are derived from two independent analytical laboratories (1 and 2). The high PBDE concentrations for the toddler and child reported by Laboratory 1 (September 2004) merited confirmation. Hence, an additional set of serum samples was collected 3 months later (December 2004) and was analyzed by Laboratory 2. Both laboratories found quite similar concentrations for the five common PBDE congeners used in the ∑ (5)PBDE (Table 1).

Both laboratories found higher PBDE serum concentrations in the children than in their parents. Concentrations of the PBDEs followed the order 18-month-old > 5-year-old > mother > father in both the September and December serum samples (Figure 1). In fact, the sum of the six major PBDEs (BDE-47, 99, 100, 153, 154, 209) in the children at both samplings matched or exceeded the 95th percentile for PBDE concentrations in U.S. women cited by McDonald (2005) as 302 ng/g lw. ∑(6)PBDEs in the 5-year-old child were 390 ng/g lw (Laboratory 1) and 258 and 251 ng/g lw (duplicate values from Laboratory 2). ∑PBDEs in the 18-month-old toddler were 651 ng/g lw (Laboratory 1) and 507 and 502 ng/g lw (Laboratory 2). These levels are uncomfortably close to body burdens associated with adverse effects on reproduction (230 ng/g lw) and neurodevelopment (660 ng/g lw) in laboratory animals (McDonald 2005).

The lower-brominated PBDEs come from a different source than BDE-209. The pentaBDE commercial flame retardant is the common source of the lower-brominated PBDEs (BDE-47, −99, −100, −153, −154), whereas the sources of BDE-209 are mainly the decaBDE commercial mixture and the octaBDE product (Bromkal 79-8DE; Chemische Fabrik Kalk, Cologne, Germany) (de Wit 2002).

Levels of BDE-209 in the children were unusually high at both sampling times and comparable to BDE-209 levels seen in work-related exposures in Sweden. For example, in September samples, BDE-209 levels (Table 1, Figure 1A) were at the high end of concentrations found in Swedish workers manufacturing decaBDE-treated rubber (Thuresson et al. 2005). In December, BDE-209 levels were 10-fold lower (Table 1, Figure 1B) but were still higher than levels observed in Swedish workers dismantling decaBDE-treated electronics (Sjödin et al. 1999). Only the December BDE-209 levels in the parents approached adult referents in Sweden (2.5 ng/g lw) (Thuresson et al. 2005).

Concentrations of BDE-209 differed significantly (10-fold) between the September and December sampling dates. This was true for all family members. The question arises, are these large differences real—that is, arising from differing exposures to decaBDE—or do they result from errors in sample collection or analysis?

We think it unlikely that the differences arise from laboratory error. Corn oil sample blanks for BDE-209 were calculated by Laboratory 1 for each of the September samples: 26 pg/g ww for the father and mother, 74 pg/g ww for the child, and 172 pg/g ww for the toddler. BDE-209 concentrations were significantly higher in all family members: Levels in the father, 5-year-old, and toddler were > 5-fold higher (124, 541, and 1,080 pg/g ww, respectively), and the mother’s level was 3-fold higher (78 pg/g ww). The relatively higher blank concentration for the mother lends some uncertainty to the precision of her September measurement, but all of the September BDE-209 values were sufficiently above background to be considered valid. The blank concentrations reported by Laboratory 2 were below the LOQ (< 0.1 ng/g lw) except for BDE-47, which was 0.1 ng/g lw.

Contamination by decaBDE during the collection of samples seems an unlikely explanation also. First, both sets of samples were collected using the same clinic, equipment, protocol, and personnel. Second, all four family members had higher BDE-209 levels in September than in December, so decaBDE contamination would have had to occur in all four samples in September. Third, indoor dust would be the likely source of contamination and is known to contain congeners from the pentaBDE and the decaBDE commercial mixtures in nearly equal proportions (Schecter et al. 2005a; Stapleton et al. 2005; Wilford et al. 2005). Consequently, if indoor dust were the source of contamination, the 10-fold higher levels of BDE-209 observed in September should have been accompanied by 10-fold higher levels of the lower-brominated PBDEs. However, only BDE-209 levels were higher.

The most likely explanation for the 10-fold difference in BDE-209 levels is the congener’s 15-day half-life in humans (Thuresson et al. 2006). As a result of its short half-life, concentrations of BDE-209 drop rapidly after reduced exposures. If the family experienced no further exposure to BDE-209 after the September sampling, BDE-209 levels 90 days later (six half-lives) would decrease significantly. Levels would drop from 233 to 3.6 ng/g lw in the toddler and from 143 to 2.2 ng/g lw in the child. BDE-209 levels measured in the toddler and child in December were higher (Table 1), suggesting some additional exposure during the 90-day period. The lower BDE-209 levels in the family in December may reflect a decrease in decaBDE exposures between sampling periods.

Only two congeners, BDE-153 and BDE-154, had higher concentrations in December than in September. For BDE-154, the higher concentrations reported by Laboratory 2 in December are likely explained by coelution with 2,2′,4,4′,5,5′-hexabromo-biphenyl (BB-153). The methodology applied by Laboratory 2 does not allow separate quantification of these two compounds, as all compounds are quantified by assessing the bromide ions *m/z* 79 and 81 (Buser 1986).

BDE-153, however, is not known to coelute with other analytes in the method applied by Laboratory 2, and BDE-153 concentrations were almost 2-fold higher in December than in September for three family members (Table 1). It is not clear what factors are responsible for these higher proportions of BDE-153. Recent studies in humans report an increasing prevalence of BDE-153 as the major congener, replacing BDE-47 (Fängström et al. 2005; Johnson-Restrepo et al. 2005; She J, unpublished data; Thuresson et al. 2006). The decreases in BDE-209 and the increases in BDE-153 we observed in family members in December may reflect metabolic debromination of BDE-209 and formation of BDE-153. Alternatively, these increases could arise from exposures to different sources of PBDEs. This needs further study.

We are left with the question: Why did the children have 2- to 15-fold higher levels than their parents of the six major PBDE congeners? A higher exposure to house dust offers one explanation. House dust contains appreciable levels of PBDEs. Indoor dust from Canadian and American homes averages 2–4 ppm PBDEs, with BDE-209 a major congener (~ 50%) (Jones-Otazo et al. 2005; Rudel et al. 2003; Schecter et al. 2005a; Stapleton et al. 2005; Wilford et al. 2005). Consequently, house dust is considered a significant source of exposure to both lower-brominated PBDEs and BDE-209. This is especially true for children: House dust may account for 80% of total daily PBDE exposure for toddlers, compared with 14% for adults (Wilford et al. 2005). Ingestion of indoor dust can lead to as much as a 100-fold higher PBDE exposure for toddlers than for adults (Jones-Otazo et al. 2005).

Indoor dust may play such a role here. We estimated exposures of family members to house dust and found the PBDE exposures paralleled their body burdens (Table 2). We estimated daily PBDE exposures from house dust on a body weight (bw) basis for the toddler, child, and parents using U.S. EPA estimates of house dust intake and the lower-brominated PBDE (∑(5)PBDE) and BDE-209 levels in house dust from studies cited.

The U.S. EPA estimates that children 1–4 years of age ingest 100 mg house dust per day, and adults ingest 50 mg per day (U.S. EPA 1993). On a bw basis, this is a 15-fold difference, as a 10-kg toddler ingests 10 mg dust/kg bw/day, whereas a 75-kg adults ingests 0.67 mg dust/kg bw/day. The house dust was estimated to contain ∑PBDEs at 2 μg/g, split evenly between ∑(5)PBDE and BDE-209 (Stapleton et al. 2005; Wilford et al. 2005). The calculations show that the estimated exposures from house dust in the toddler and child are 15- and 7.5-fold higher than in the parents, reasonably similar to the ratios of their body burdens of BDE-209 (Table 2). This agrees with the prediction made by Jones-Otazo et al. (2005) that younger children have higher body burdens of PBDEs and see greater contributions from house dust.

Diet is another potential source of exposure. The lack of U.S. data on dietary PBDE levels hampers dietary exposure estimates for PBDEs. However, there are good data on breast milk (Schecter et al. 2005b; She et al., in press), an exposure unique to the toddler.

We estimate that human milk contributes significantly to the higher levels of the lower-brominated PBDEs observed in the toddler, as predicted by Jones-Otazo et al. (2005) and as seen with other environmental pollutants such as polychlorinated dibenzo-*p*-dioxins (Abraham et al. 1996). For BDE-209, however, breast milk is a minor contributor to the toddler’s higher levels compared with house dust (Table ^2).^ ∑(5)PBDE levels in California and the Pacific Northwest average about 50 ng/g lw (She et al., in press). BDE-209 is a minor congener in human milk, averaging about 1 ng/g lw (Schecter et al. 2003, 2005b; She et al., in press). Human milk averages about 4.5% lipid, with a 12-month-old child ingesting 425 mL human milk/day (U.S. EPA 1996). For the toddler, we estimate that breast milk is a significant source for ∑(5)PBDE, whereas house dust is a significant source for BDE-209 (Table 2).

## Conclusion

Our case study underscores the absence of data on PBDE body burdens in children. We identified two young children with PBDE concentrations at or above the 95th percentile for U.S. adults. BDE-209 levels in these children were comparable to levels found in occupationally exposed workers in Sweden and were an order of magnitude higher than BDE-209 levels in their parents at both time points. Our results suggest three hypotheses that deserve further study:

- Children have higher PBDE concentrations than adults, concentrations that may be high enough to cause harm.
- House dust is a more significant source of PBDE exposures in children than in adults. This is particularly true for exposure to BDE-209, the major PBDE congener in house dust.
- Body burdens of BDE-209 in children and adults can decrease rapidly in response to decreased exposures.

Given the widespread use of PBDEs in society and the vulnerability of children to the effects of environmental chemical exposures, we see great need to measure and characterize PBDE levels in the very young.

## Figures and Tables

**Table 1:** PBDE concentrations (ng/g lw) in September and December (duplicate) 2004 serum samples.

Sample	Date	Lipid (%)	BDE-47	BDE-99	BDE-100	BDE-153	BDE-154^a^	∑(5)PBDE	BDE-209
Father	Sept	0.42	32	4	8	19	0.6	64	23
	Dec	0.42	29;23	5;5	11;10	25;24	5;5	75;67	2;3
Mother	Sept	0.38	60	10	13	22	1.3	106	14
	Dec	0.37	62;52	16;16	22;21	41;42	6;6	147;137	4;4
Child	Sept	0.33	137	28	30	49	3	247	143
	Dec	0.46	105;94	34;34	39;39	64;65	7;7	249;239	9;12
Toddler	Sept	0.39	245	37	57	75	4	418	233
	Dec	0.31	209;186	45;45	86;87	132;141	16;17	488;476	19;26

a Data from December (Laboratory 2) may include polybrominated biphenyl PBB-153.

**Table 2:** Comparison of estimated exposures to ∑(5)PBDE^a^ and BDE-209 from indoor dust and breast milk with ∑(5)PBDE and BDE-209 concentrations among family members.

	Indoor dust (est ng/kg bw/day)		Breast milk (est ng/kg bw/day)		Body burdens (measured, normalized to parents)		
	∑(5)PBDE	BDE-209	∑(5)PBDE	BDE-209	∑(5)PBDE	BDE-209 (Sept)	BDE-209 (Dec)
Toddler	10^b^	10^c^	94^d^	2^e^	9	13	7
Child	5	5	—	—	5	8	3
Parents (avg)	1^f^	1^g^	—	—	2	1	1

Abbreviations: —, no data; avg, average; est, estimated.

a BDE-47, BDE-99, BDE-100, BDE-153, BDE-154.

b (1,000 ng ∑(5)PBDE/1 g dust) (0.1 g dust/1 day) (1/10 kg) = 10 ng/kg bw/day.

c (1,000 ng BDE-209/1 g dust) (0.1 g dust/1 day) (1/10 kg) = 10.

d (50 ng ∑(5)PBDE/1 g lw) (0.045 g lw/1 g milk) (425 g milk/1 day) (1/10 kg) = 94.

e (1 ng BDE-209/1 g lw) (0.045 g lw/1 g milk) (425 g milk/1 day) (1/10 kg) = 2.

f (1,000 ng ∑(5)PBDE/1 g dust) (0.05 g dust/1 day) (1/75 kg) = 0.67.

g (1,000 ng BDE-209/1 g dust) (0.05 g dust/1 day) (1/75 kg) = 0.67.
